# Forecasting emergency department arrivals using INGARCH models

**DOI:** 10.1186/s13561-023-00456-5

**Published:** 2023-10-28

**Authors:** Juan C. Reboredo, Jose Ramon Barba-Queiruga, Javier Ojea-Ferreiro, Francisco Reyes-Santias

**Affiliations:** 1 Department of Economics, University of Santiago (USC), Santiago de Compostela, Spain; 2 ECOBAS Research Centre, Santiago de Compostela, Spain; 3 EOXI Santiago de Compostela, SERGAS, Santiago de Compostela, Spain; 4 Bank of Canada, 234 Wellington Street, Ottawa, ON K1A 0G9 Canada; 5 Departamento de Organización de Empresas y Marketing, Universidad de Vigo. Facultad de Ciencias Empresarias e Turismo, Campus Universitario s/n, As Lagoas, 32004 Spain; 6 IDIS, Santiago de Compostela, Spain

**Keywords:** Emergency department, Forecasting, Patient arrivals, INGARCH models

## Abstract

**Background:**

Forecasting patient arrivals to hospital emergency departments is critical to dealing with surges and to efficient planning, management and functioning of hospital emerency departments.

**Objective:**

We explore whether past mean values and past observations are useful to forecast daily patient arrivals in an Emergency Department.

**Material and methods:**

We examine whether an integer-valued generalized autoregressive conditional heteroscedastic (INGARCH) model can yield a better conditional distribution fit and forecast of patient arrivals by using past arrival information and taking into account the dynamics of the volatility of arrivals.

**Results:**

We document that INGARCH models improve both in-sample and out-of-sample forecasts, particularly in the lower and upper quantiles of the distribution of arrivals.

**Conclusion:**

Our results suggest that INGARCH modelling is a useful model for short-term and tactical emergency department planning, e.g., to assign rotas or locate staff for unexpected surges in patient arrivals.

## Background

The hospital emergency department (ED) is the basic unit providing an immediate response to emergency health problems. It is a core component in any health system in providing care for urgent and potentially serious pathological processes with a possible outcome of death or requiring immediate diagnosis and treatment to avoid pain. ED activity is both intense and very diverse, covering from immediately life-threatening pathologies (e.g., cardiorespiratory arrest) to serious or potentially serious illnesses requiring diagnosis or treatment in the hospital setting (e.g., polytrauma, acute myocardial infarction). EDs additionally deal with less serious emergencies that may require hospitalization for diagnosis (e.g., retinal detachment, pyelonephritis) and also provide initial treatment and observation without necessarily involving admission. EDs also serve around 40–50% who could feasibly be treated in primary care emergency centres or 24-h emergency care facilities with an intermediate resolution. About 15% of the population (elderly, frail or chronically ill patients) use hospital ED services on a recurring basis as a result of the aggravation of their pathologies [14, 29].

Patient arrival in EDs is uneven over time. Distribution over the days of the week is not uniform and, although there are variations from one centre to another, some days account for a clearly higher number of visits, e.g., Mondays (see [9, 20]. Likewise, distribution throughout the year is not uniform. Demand for care varied in relation to holiday periods (demographic movements), respiratory virus epidemics, climatic and atmospheric changes and social events [20]. Handling surges, which is the main challenge to ensuring efficient ED management and functioning [7, 16, 21, 26], is closely related to appropriate timing of treatment. ED and hospital resources therefore have to be planned with some built-in flexibility in order to adapt to changing and cyclical changes in the demand for services.

In addition to the quantitative aspects of patient arrivals in EDs, there is a great qualitative impact, given that ED diagnostic and therapeutic activities determine the subsequent evolution of admitted patients in terms of illness resolution, including length of stay, complications and patient satisfaction. Patient satisfaction/dissatisfaction with healthcare services in general is strongly conditioned by technical quality and, above all, by perceived ED quality, which determines perceptions of overall hospital performance [11].

To avoid congestion and facilitate appropriate delivery of medical services, efficient management of ED services requires accurate forecasting of patient inflows [3, 5, 8, 25, 31]. Forecasting is challenging, however, as daily and seasonal variations in patient arrivals are featured by a high degree of variability and overdispersion [20, 23]. Previous empirical research has extensively explored the dynamics of arrivals, mainly relying on Poisson and negative binomial models with different extensions (see, e.g., [3, 28, 34, 35]. However, whether arrivals can be predicted from both past mean values and past observations is still an open question. Nonetheless, information on past mean values and past observations could be useful not only to make accurate mean value forecasts, but also to make predictions at the lower and upper arrival distribution quantiles, critical for two reasons: (a) efficient healthcare resource allocation when patient arrival numbers are low, and (b) avoidance of the negative impact of patient overflows on healthcare quality.

The objective of this study is to explore whether past mean values and past observations are useful to forecast daily patient arrivals in an ED, using an integer-valued generalized autoregressive conditional heteroscedastic (INGARCH) model. This model was designed to describe integer-value series featured by small values and overdispersion, not appropriately addressed by autoregressive moving average (ARMA) models. Originally proposed by Grunwald et al. [15] and Heinen [17], an INGARCH process has a Poisson or a negative binomial conditional distribution, with a time-varying intensity parameter given by a linear function of its p-lagged values and its q recent observations. Sharing the spirit of generalized autoregressive heteroscedastic (GARCH) models, an INGARCH model, by efficiently using past patient inflow information and reflecting the dynamics of the volatility of arrivals, can potentially yield a better conditional distribution fit and forecast for patient arrivals.

Our empirical study focuses on daily arrivals, as this frequency is useful for both routine planning (e.g., on rotas) and tactical planning (e.g., decisions on contacting staff). Daily forecasts provide useful support for administrative decision making and gives early warning signals to efficiently handle available physical and human resources. Our empirical results for a large hospital conclude that the INGARCH model improves both in-sample and out-of-sample forecasts. In particular, the INGARCH model yields a better fit both in the mean and the tails of the arrival distribution, and thus reports more accurate forecasts for abrupt upward or downward movements in arrivals.

Our empirical evidence has implications to support ED management and planning decisions, given that our modelling approach provides more accurate predictions than those based on average counts of patient arrivals, in that it reflects all available past information. Our forecasting model is especially useful when patient inflow is intense, as this is when efficient resource allocation is critical to delivering healthcare quality. Given that ED arrival data is structured similarly across different hospitals, our evidence could be considered generalizable to other hospital EDs.

The paper is organized as follows: Material and Methods and Results sections describe the models and the data, respectively, and Discussion section presents and discusses the empirical results. Finally, Conclusion section concludes the paper.

## Material and methods

### The INGARCH model

To account for past mean values and past observations regarding ED arrivals, we could adapt the count data nature of ED arrivals to a transformation, e.g., using a logarithmic transformation, and then use standard estimation procedures. However, this modelling strategy to deal with count data has several drawbacks regarding inference and negative predicted values [36], described and summarized in Table 1.

**Table 1:** Models for non-count data adapted for count data and their limitations

Model	Advantages	Disadvantages
Normal linear regression $y=x\beta +\epsilon$ $\epsilon \sim N\left(0,{\sigma }^{2}\right)$	Normal distribution approximates the Poisson distribution if the mean is higher than 20	No possible inference on single outcomes The model allows for a negative outcome The prediction is not coherent, i.e., the forecast is not an integer-valued outcome
Log-linear model $\mathrm{log}\left(y\right)=x\beta +\epsilon$ $\epsilon \sim N\left(0,{\sigma }^{2}\right)$	The variable y is modelled as a log-normal variable	The zeros in the data have to be deleted to estimate this model, which leads to endogenous sample selection problems The prediction is not coherent, i.e., the forecast is not an integer-valued outcome There is a restriction on the conditional variance, i.e., it must be quadratic in the conditional expectation.*
Log-linear model with constant c to deal with zeros $\mathrm{log}\left(y+c\right)=x\beta +\epsilon$ $\epsilon |x\sim N\left(0,{\sigma }^{2}\right)$	The model can be estimated even if there are zero elements in the dataset	The log(y) is not linear in x, which introduces bias in the estimation of the model The prediction is not coherent, i.e., the forecast is not an integer-valued outcome
Non-linear model $y=\mathrm{exp}\left(\mathrm{x\beta }\right)+\upepsilon$ $\epsilon \sim N\left(0,{\sigma }^{2}\right)$	There is no problem in dealing with zero values	The model allows for a negative outcome The prediction is not coherent, i.e., the forecast is not an integer-valued outcome
Ordered probit and logit state equation: ${y}^{*}=x\beta +\epsilon$ Observation equation: $y=0\;\text{if}\;{y}^{*}<{\alpha }_{0}$ $y=1\;\text{if}\;{\alpha }_{0}\le {y}^{*}<{\alpha }_{1}$ $y=2\;\text{if}\;{\alpha }_{1}\le {y}^{*}<{\alpha }_{2}$ $\vdots$	The integer-valued structure of the data is considered The prediction can be coherent, i.e., if we wanted to forecast the future median value, it would be an integer-valued outcome	The underlying count process is not reflected The forecast is limited to values already observed in the data Complexity is excessive when the number of counts is high

^*^If a variable y follows a log-normal distribution, the following identity holds: ${\varvec{V}}{\varvec{a}}{\varvec{r}}\left({\varvec{y}}|{\varvec{x}}\right)=\left({{\varvec{e}}}^{{{\varvec{\sigma}}}^{2}}-1\right){\left[{\varvec{E}}\left({\varvec{y}}|{\varvec{x}}\right)\right]}^{2}$

In this research we use the INGARCH model, which is the integer-valued counterpart to the conventional GARCH model [33], where the IN indicates the integer-valued structure of the data [32]. This model is also referred to as the autoregressive conditional Poisson model [18] or the Poisson autoregressive model [13].

A count variable ${Y}_{t}$ follows an INGARCH(p, q) model if its conditional Poisson distribution has a conditional mean ${\lambda }_{t}$ as given by the following recursion:

1 $${\lambda }_{t}=\omega + \sum\nolimits_{i=1}^{p}{\alpha }_{i}{Y}_{t-i}+\sum\nolimits_{j=1}^{q}{\beta }_{j}{\lambda }_{t-j}$$

where $\omega >0$ and ${\alpha }_{1}, \dots , {\alpha }_{p},{\beta }_{1}, \dots ,{\beta }_{q}\ge 0$ and $\sum_{i=1}^{p}{\alpha }_{i}+\sum_{j=1}^{q}{\beta }_{j}<1$ for stationarity reasons [10]. Thus, the conditional Poisson distribution evolves over time with a mean parameter that depends on its previous values and on the past values of the studied variable. This distribution is, therefore, conditional equidispersed but unconditional overdispersed.

For the particular case of an INGARCH(1,1) model (see [10, 19], we have $E\left({Y}_{t}|{Y}_{t-1}\right)={\lambda }_{t}=Var\left({Y}_{t}\right|{Y}_{t-1})$. Applying the law of iterated expectations it follows that $E\left({Y}_{t}\right)=E\left(E\left({Y}_{t}|{Y}_{t-1}\right)\right)=E\left({\lambda }_{t}\right)=\frac{\omega }{1-\alpha -\beta }$. Finally, using the law of total variance, it follows that $Var\left({Y}_{t}\right)=E\left(Var\left({Y}_{t}|{Y}_{t-1}\right)\right)+Var\left(E\left({Y}_{t}|{Y}_{t-1}\right)\right)=E\left({\lambda }_{t}\right)+Var\left({\lambda }_{t}\right)>E\left({\lambda }_{t}\right)$ and $Var\left({\lambda }_{t}\right)=\frac{1-{\left(\alpha +\beta \right)}^{2}+{\alpha }^{2}}{1-{\left(\alpha +\beta \right)}^{2}}E\left({\lambda }_{t}\right)$.

The INGARCH model enables a long memory process to be modelled parsimoniously, where the conditional mean depends on the whole history of the process. For the particular case of the INGARCH(1,1), we have [12]:

2 $${\lambda }_{t}=\alpha \sum\nolimits_{k=1}^{t}{\beta }^{k-1}{Y}_{t-k}+{\beta }^{t}{\lambda }_{0}+\omega \frac{1-{\beta }^{t}}{1-\beta },$$

where ${\lambda }_{0}$ could be estimated as an additional parameter [10].

Alternative specifications to the Poisson INGARCH model are the negative binomial INGARCH model, where the recursion in Eq. (1) refers to $\mathrm{log}\left({\lambda }_{t}\right),$ the non-linear Poisson autoregression and a model that includes the covariate information in Eq. (1) [1]. The main advantage of assuming a negative distribution instead of a Poisson distribution lies in the greater flexibility, as the variance may be larger than the mean. Indeed, in the Poisson model we have ${\lambda }_{t}={\mu }_{t}={\sigma }_{t}^{2}$, while for the negative binomial model we have ${\sigma }_{t}^{2}=\frac{{\mu }_{t}}{\pi }=\frac{\nu \left(1-\pi \right)}{{\pi }^{2}}$ and, depending on the model specification, the dynamics are set in $\pi$ [38] or in $\nu$ [37]. Interestingly, the Poisson could be seen as a particular case of the negative binomial when $\pi \to 1$ and $\nu \to \infty$.

For the case of the negative binomial distribution, $NB(\nu , \pi )$ with $\nu >0$ and $0<\pi <1$, the time-varying parameter $\pi$ is modelled via the equation ${\pi }_{t}=\frac{1}{1+\frac{{\lambda }_{t}}{\nu }}$ [38], and the dynamics of the parameter $\nu$ through the equation ${\nu }_{t}=\frac{{\lambda }_{t}\pi }{1-\pi }$ [37].^1^ The probability distribution mass of $Y$, where $Y\sim NB(\nu , \pi )$ is

3 $$P\left(Y=y\right)={\left(1-\pi \right)}^{y}{\pi }^{\nu }\left(\begin{array}{c}y+\nu -1\\ \nu \end{array}\right).$$

The parameters of those models are estimated by maximum likelihood, where the objective function is given by $\sum_{t=1}^{T}\mathrm{log}\left(P\left(Y=y|{\theta }_{t}\right)\right)$, with ${\theta }_{t}={\lambda }_{t}$ for the Poisson distribution (see Eq. (1)), ${\theta }_{t}=\left({\pi }_{t},\nu \right)$ for the negative binomial model by Zhu [38], and ${\theta }_{t}=\left(\pi ,{\nu }_{t}\right)$ for the negative binomial by Xu [37].

To evaluate forecast accuracy, we use the mean squared error (MSE) and the mean absolute error (MAE), which compare the mean and median, respectively, with the real number of arrivals. The MSE is computed as:

4 $$MSE=\frac{1}{T-k}\sum\nolimits_{t=k}^{T}{\left({y}_{t}-{\overline{y} }_{t|t-1}\right)}^{2},$$

where ${y}_{t}$ denotes patient arrivals at time t, and ${\overline{y} }_{t|t-1}$ is the mean number of patient arrivals at time t forecasted with the data available at time t-1. The MAE is computed as:

5 $$MAE=\frac{1}{T-k}\sum\nolimits_{t=k}^{T}{|y}_{t}-{\widetilde{y}}_{t|t-1}|,$$

where ${\widetilde{y}}_{t|t-1}$ is the median of the patient arrival distribution at time t, built using the data obtained at t-1.

In addition, to evaluate the fit of the future entire distribution with respect to real patient arrival data, we compute the probability integral transformation (PIT) [6, 22]. Relative frequencies are obtained as the ratio between the forecasted PIT of two consecutive quintiles and the probability of a perfect data fit,the closer the bars to one, the better the fit of forecasted values. Consecutive quintiles are given by $\widetilde{F}\left(\frac{j}{10}\right)-\widetilde{F}\left(\frac{j-1}{10}\right)$ for j = 1,…,10, where:

6 $$\widetilde{F}\left(u\right)=\left\{\begin{array}{cc}\begin{array}{c}0 \\ \frac{u-F\left(k-1|{I}_{t-1}\right)}{F\left(k|{I}_{t-1}\right)-F\left(k-1|{I}_{t-1}\right)}\\ 1 \end{array}& \begin{array}{c}u\le F\left(k-1|{I}_{t-1}\right)\\ F\left(k-1|{I}_{t-1}\right)\le u\le F(k|{I}_{t-1})\\ u\ge F\left(k|{I}_{t-1}\right)\end{array}\end{array}\right.$$

where $k>0$ and $F(\cdot )$ is the predictive distribution.

We also include a threshold that does not reject the null hypothesis of the data coming from a uniform (0,1) distribution. Intervals are created similarly to the threshold of a backtesting exercise. We assume that each observation has 1/10 probability of being in each bar, so the distribution of the observations in the PIT histogram reflects a bin (T, 0.1), where T indicates the number of out-of-sample observations. This technique allows us to check whether the data structure is fitted correctly in short sample series. To our knowledge, there is no previous study of count models that uses a statistical criterion for small samples to evaluate the PIT histogram.

### Data

**Fig. 1:**
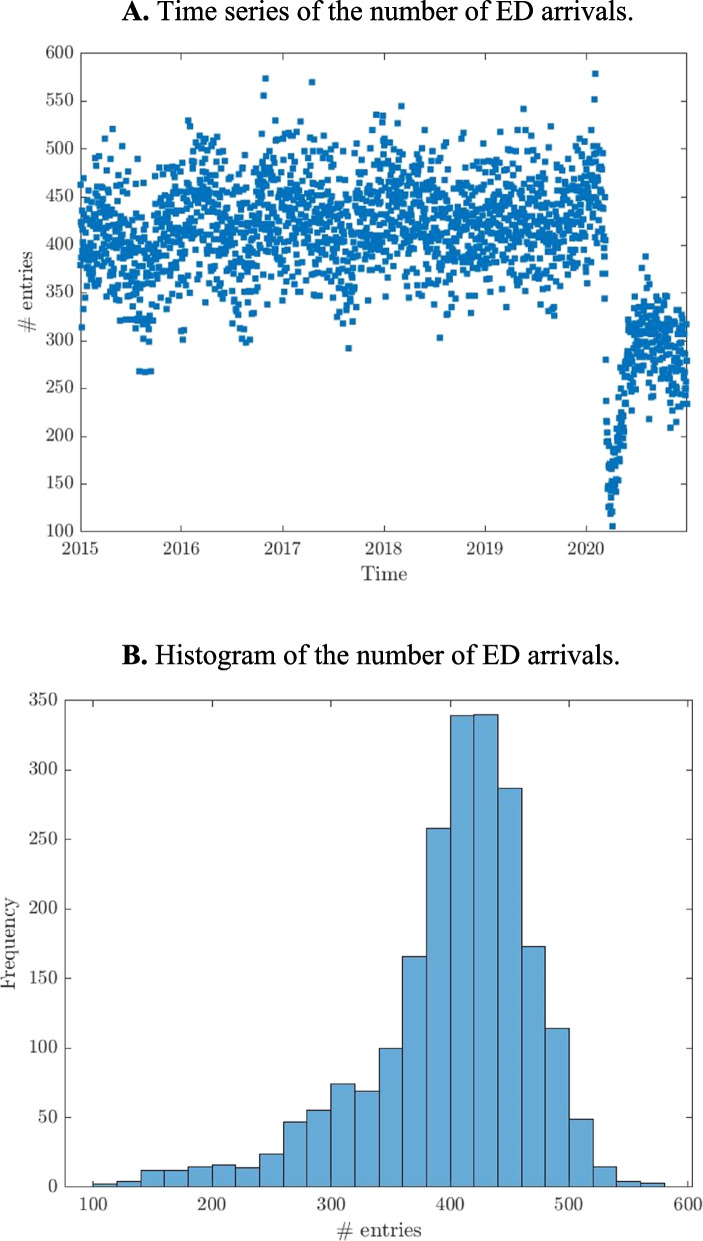
Time series and histogram of the number of ED arrivals. **A** Time series of the number of ED arrivals. **B** Histogram of the number of ED arrivals

The data corresponds to daily arrivals in the ED of a large 1100-bed university clinical hospital in Santiago de Compostela (Spain) during January 2015 to December 2020, with a catchment population of about 450,000 people in that period. ED human resources include 36 doctors, 57 nurses and 42 clinical assistants, while physical resources include 21 reclining chairs, a critical room with four monitored stations for vital emergencies and a monitor room with six monitored stations for serious emergencies or patients requiring monitored observation. The ED applies Manchester triage, which classifies and colour codes patients into five levels according to urgency. Of the patients who attend the ED, 22.04% are admitted to hospital wards, 77.1% are discharged home, 0.48% are transferred to another hospital, 0.21% die in the ED and 0.17% request voluntary hospital discharge. Figure 1A shows that inflow seemed to show a seasonal trend, but was at a minimum during the early COVID crisis, as reflected in the long left tail in the histogram in Fig. 1B, and as reflected in the negative skewness reported in Table 2. Before the COVID pandemic, the mean number of daily arrivals was around 400, but structural change since then has reduced that number to around 300, and the mean number of monthly and annual arrivals is 12,207 and 146,483, respectively. Table 2 shows that since the variance is much larger than the mean, a model that takes into account this overdispersion is required, e.g., a negative binomial model. The fact that the number of entries is far from zero also indicates that zero-inflated models should be ruled out.

**Table 2:** Four first moments of the number of ED arrivals for the period 2015–2020

	2015–2020	2015–2019	2019–2020
mean	400.96	419.01	364.81
variance	4836.52	2017.46	8530.98
skewness	-1.17	-0.05	-0.54
kurtosis	4.90	3.11	2.49

**Fig. 2:**
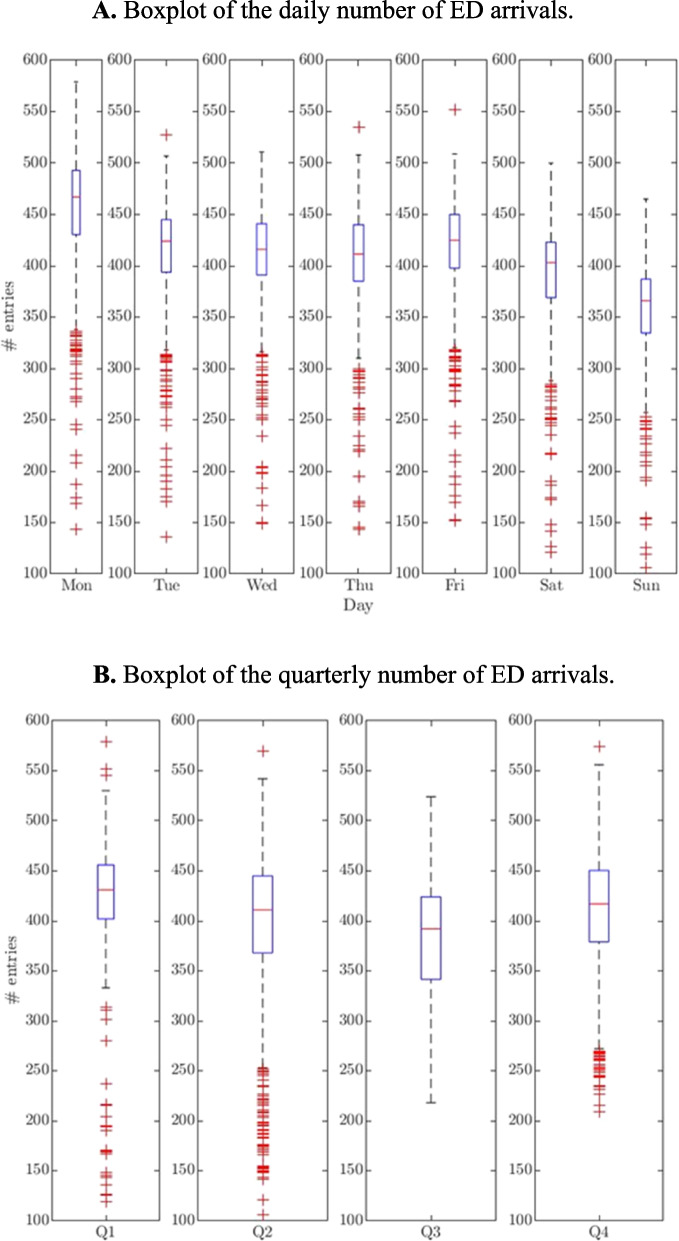
Boxplots of the number of ED arrivals. **A** Boxplot of the daily number of ED arrivals. **B** Boxplot of the quarterly number of ED arrivals

Figure 2 depicts trends that need to be considered when modelling the number of arrivals. Figure 2A indicates that the Monday arrival rate is considerably greater than that of the remaining weekdays, whereas the weekend rate is much lower than the workday rate. Figure 2B depicts a higher number of arrivals in the first (spring) and fourth (winter) quarters compared to the second (summer) and third (autumn) quarters of the year.

## Results

### Empirical evidence

Taking into account the features of daily ED arrivals, we use the INGARCH model as it allows changes in the count distribution to be captured by considering changes in the mean, as reported by Fig. 1 Panel A. Specifically, we consider an INGARCH(1,1) model with a negative binomial distribution to capture the overdispersion in the data, i.e., the variance is higher than the mean, as reported in Table 2. Furthermore, we use deterministic covariates to identify the increase in arrivals on Mondays and in the first and fourth quarter, and the decrease in arrivals at weekends and after the COVID outbreak. To avoid overfitting problems, we keep model parameterization to a minimum. Equation (7) presents the evolution of the conditional mean of a negative binomial model, i.e., the Zhu [38] and Xu [37] model specifications, or the conditional mean of a Poisson model.^2^ Hence $\mathrm{X}=[{I}_{Monday}, {I}_{Weekend}, {I}_{Winter}, {I}_{COVID}]$, and thus:

7 $${\lambda }_{t}=\omega + \alpha {Y}_{t-1}+\beta {\lambda }_{t-1}+\gamma {X}_{t|t-1}.$$

Given that $E\left({\lambda }_{t}\right)=\frac{\omega }{1-\alpha -\beta }$, in order to have comparable estimates for the exogenous variables in Eq. (7) across different model specifications, we set the parameter $\omega$ to be equal to $(1-\alpha -\beta$) multiplied by the mean number of arrivals, computed by discarding the dates that are considered within the exogenous variables, i.e., the mean number of arrivals on days that are not Monday, the weekend, or winter (Q4), or after the COVID outbreak (after 13 March 2020, when the Spanish government declared a state of alarm).

**Fig. 3:**
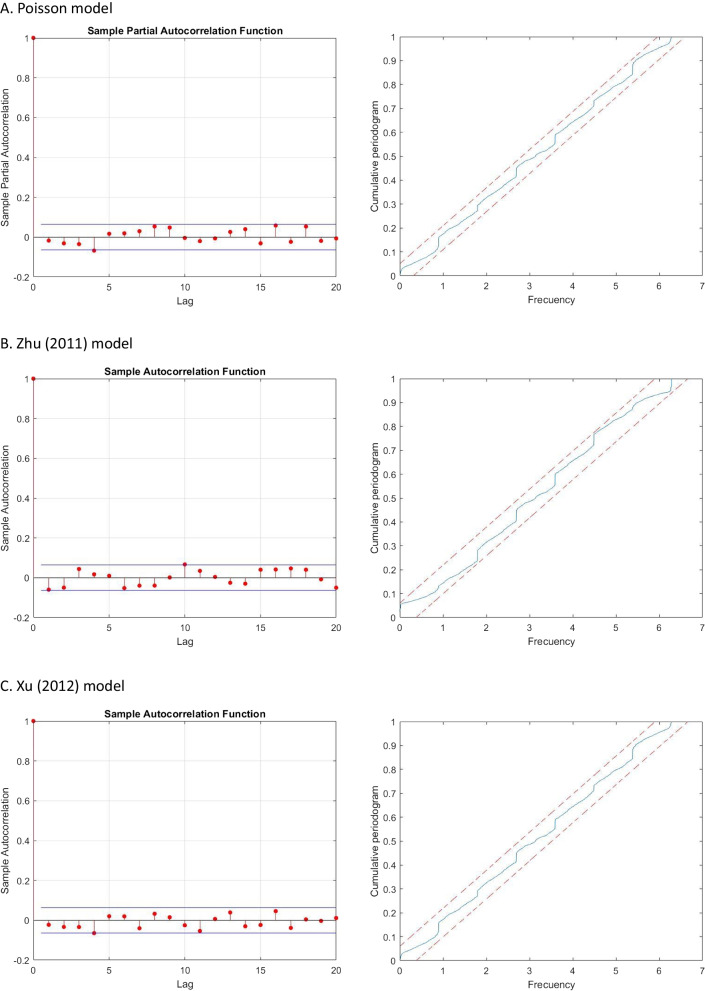
Autocorrelation functions and cumulative periodograms of the Pearson residuals. **A** Poisson model. **B** Zhu [38] model. **C **Xu [37] model

Table 3 presents the parameters of the Zhu [38], Xu et al. [37] and Poisson models for $NB(\nu , \pi )$, where parameter $\theta$ in Table 2 refers to parameter $\nu$ for Zhu [38] and to parameter $\pi$ for Xu et al. [37]. Empirical estimates show that, consistent with the above-mentioned descriptive features of the data, the Monday effect is positive and significant, while the weekend effect and the winter effect are both negative and significant. Finally, the COVID pandemic had a negative impact on mean ED arrivals, consistent with the fall in hospital activity except for COVID pathologies. Estimates of the AR and MA parameters show that those effects are positive and statistically significant, indicating that both past mean values and past observations are useful in depicting the conditional distribution of patient arrivals and, thus, in forecasting those arrivals. This evidence holds for both the Zhu [38] and the Xu et al. [37] model specifications. Finally, we obtain the Pearson residuals for all the different model specifications and compute the autocorrelations and the cumulative periodogram, confirming that those residuals are white noise as shown in Figs. 3.

**Table 3:** Parameters estimates and standard deviation (in parenthesis) for the model in Eq. (7)

	$\mathrm{\alpha }$	$\beta$	$\theta$	${\gamma }_{Winter}$	${\gamma }_{Monday}$	${\gamma }_{Weekend}$	${\gamma }_{COVID}$	LogLik	AIC	BIC
Zhu [38]	0.27 ***	0.68 ***	235.14 ***	-0.00 ***	0.18 ***	-0.07 ***	-0.03 ***	-10,726	21,467	21,507
	(0.01)	(0.01)	(0.01)	(0.01)	(0.01)	(0.01)	(0.01)			
Xu et al. [37]	0.26 ***	0.67 ***	0.37 ***	0.37 **	0.17 ***	-0.07 ***	-0.03 ***	-10,723	21,461	21,500
	(0.01)	(0.01)	(0.01)	(0.00)	(0.01)	(0.00)	(0.00)			
Poisson	0.18 ***	0.77 ***		-0.00 ***	0.17 ***	-0.07 ***	-0.02 ***	-11,537	23,086	23,120
	(0.01)	(0.01)		(0.00)	(0.00)	(0.00)	(0.00)			

***, **, and * indicate that the parameter is significant at 1%, 5% and 10%, respectively. The parameters are estimated using the full sample. Parameter $\omega$ in Eq. (7) is obtained by weighting, by (1 $-\mathrm{\alpha }-\upbeta$), the mean of the number of arrivals on days not affected by the dummies

**Fig. 4:**
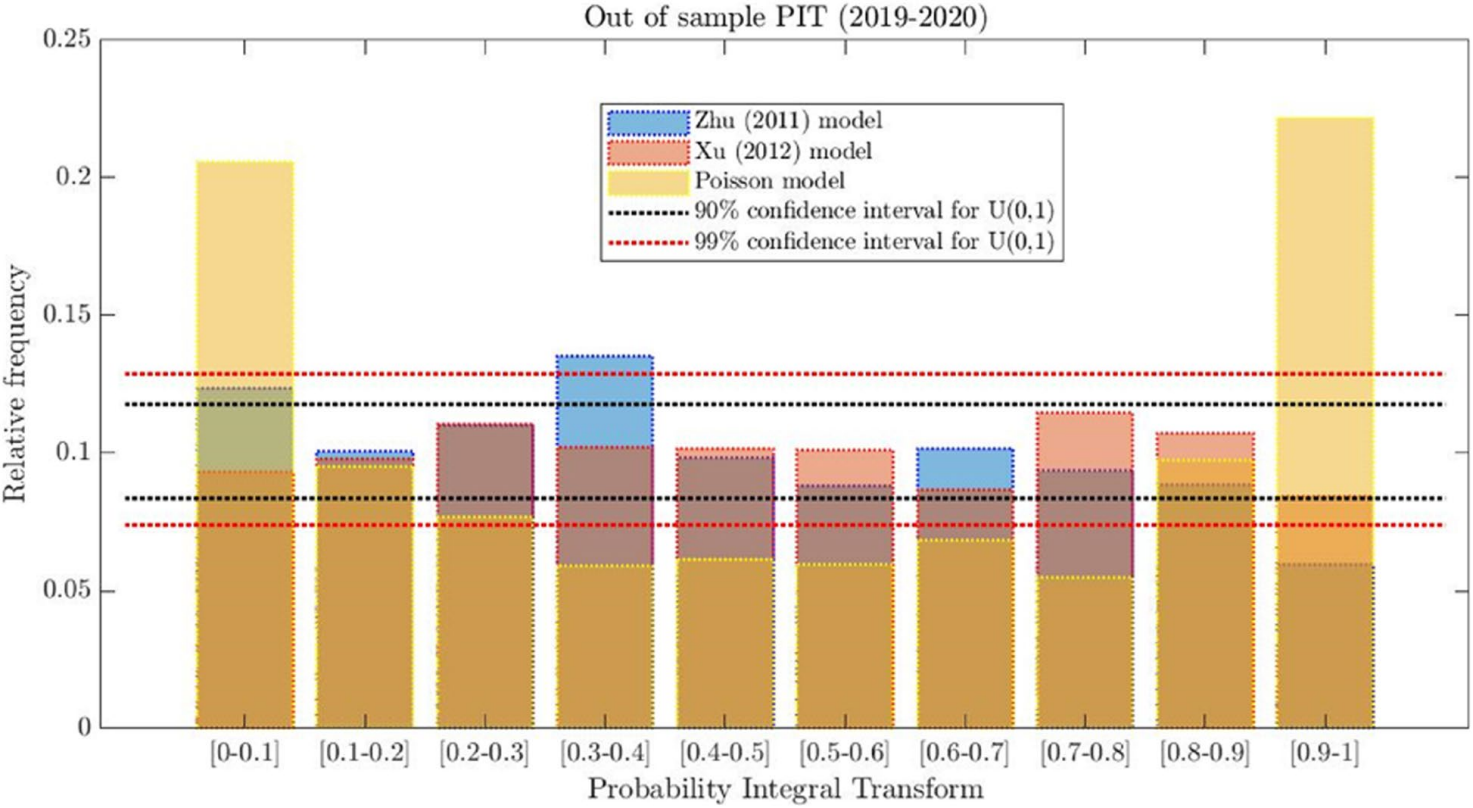
Probability integral transformation (PIT) for the out-of-sample period 2019–2020

Figure 4 depicts the PIT for the Zhu [38], Xu et al. [37] and Poisson models, showing that all the bars from Xu et al. [37] are within the red lines (indicating the null hypothesis of being uniformly distributed at 99%), but not those for the Zhu [38] model. Interestingly, the Poisson PIT is U-shaped, indicating that the restriction imposed by this model, i.e., the conditional mean equals the conditional variance, results in a failure to forecast lower and upper quantiles of the distribution of arrivals.

Finally, to mitigate concerns on overfitting, we run an out-of-sample evaluation for a rolling window of all the data prior to the day we want to forecast. Results for the comparison of each model in terms of the one-day forecast of the number of arrivals are reported in Table 4, which shows the in-sample (2015–2018) and out-of-sample (2019–2020) evidence for those metrics. Empirical estimates show that the Xu et al. [37] model yields lower MSE and MAE values for both the in-sample and out-of-sample periods. In addition, Table 5 shows that, according to Pearson’s residual autocorrelation, the Zhu [38] model also yields a good fit for the out-of-sample period.

**Table 4:** In-sample and out-of-sample MSE and MAE for ED arrivals

	MSE		MAE	
	In-sample (2015–2018)	Out-of-sample (2019–2020)	In-sample (2015–2018)	Out-of-sample (2019–2020)
Zhu [38]	1251.28	1020.70	27.74	25.16
Xu et al. [37]	1102.80	979.06	25.91	24.52
Poisson	1102.00	981.08	25.94	24.58

The one-day-ahead forecast is estimated using the information available up to the previous day

**Table 5:** Pearson’s autocorrelation from the raw data and the residuals from the models in the out-of-sample periods (2019–2020)

	Raw data	Zhu	Xu	Poisson
correlation	0.85	-0.03	-0.07	0.03
*p*-value	0.0000	0.4863	0.0597	0.2103

**Fig. 5:**
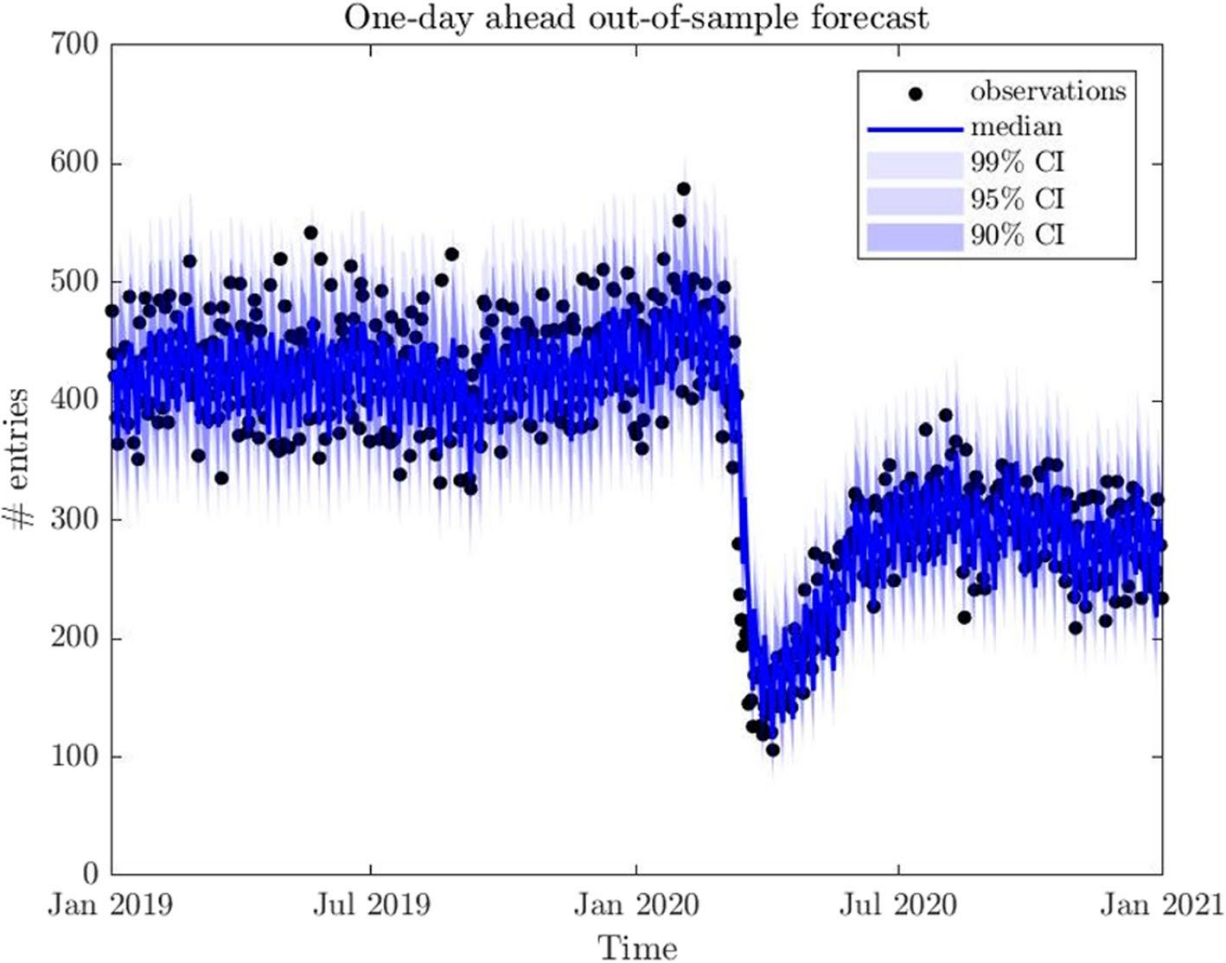
One-day-ahead out-of-sample forecast for the number of ED arrivals during 2019–2020

Figure 5 shows the one-day-ahead out-of-sample forecast of the number of arrivals in the out-of-sample period 2019–2020 using the Xu [37] model, given that this is the best forecaster. The solid line indicates the median and the dots reflect the observations (i.e., arrivals), while the different shades of blue reflect confidence intervals at different levels. Graphical evidence confirms the goodness of the model forecasting capacity.

## Discussion

Our evidence has clear implications in terms of cost minimization, as better predictions at the tails of the ED arrival distribution contribute to reduced costs through better workforce management for different circumstances. Likewise, a better analysis of ED patient arrivals allows timely care without delays, leading to improved survival rates, reduced average hospital stay and reduced readmissions of patients admitted to the ED, all of which economically translates into cost reductions.

Our evidence is related with previous studies as follows. To forecast waiting times in an emergency department, Benevento et al. [4] evaluate several machine learning techniques, including Lasso, Random Forest, Support Vector Regression, Artificial Neural Network and Ensemble methods. They define as additional predictors new variables based on the queues, which captured the situation of hospital emergencies, and show that Random Forest is a reasonable compromise solution. Our study adds to this analysis by exploring how the INGHARCH model is able to capture the dynamics of arrivals to a hospital emergency department.

Similarly, Loureiro et al. [24] evaluate the application of the CRISP-DM (Cross-Industry Standard Process for Data Mining) methodology to a demonstration case of queue waiting time prediction, with the objective of studying a machine learning (ML) method for estimating queue waiting time. The computational experiments were based on two main validation procedures: a standard cross-validation and a sliding window scheme. Overall, competitive and quality results were obtained using an AutomatedML (AutoML) algorithm fed with newly engineered features. In fact, the AutoML model proposed by the authors produces a small error (5 to 7 min), while requiring a reasonable computational effort. With less computational effort, the model presented in the current paper allows a data fit whose result does not differ too much from the one presented by the aforementioned Loureiuro et al.

von Wagner et al. [30] show how to accurately and automatically characterize patient flow in an emergency department using a combination of data from a real-time locating system (RTLS) and other traditional hospital information systems, such as electronic medical records (EMR) and laboratory information systems. The hospital can use the information to identify bottlenecks and to develop strategies to optimize patient flows. Those authors used different performance indicators, such as total length of stay, to assess Emergency Department time tasks, which is consistent with our study. One of their main conclusions is that there is a large difference between length of stay using only electronic medical record data and that calculated by combining data from electronic medical records and real-time location systems; a limitation we also found in our study.

Overall, our results suggest that INGARCH modelling is a useful support for short-term ED planning to assign rotas or locate staff for unexpected surges in patient arrivals. Improved forecasting of ED arrivals is a first step to implementing useful real-time management algorithms that offer solutions to complex ED management, in terms of both resource use and health implications for patients. Furthermore, better forecasting of ED arrivals is useful to predict hospital admissions and the impact of ED arrivals on bed utilization and length of stay [27]. However, a task we leave for future research is how ED arrivals and their forecast through INGARCH models could ultimately shape hospital admissions and bed utilization.

## Conclusions

Hospital EDs experience fluctuating and sometimes unexpected demand pressures, which complicates the efficient deployment of resources and potentially affect the quality of healthcare provision. Therefore, modelling and forecasting ED arrivals is critical to deal with inflows to EDs. The usefulness of INGARCH models to predict daily ED arrivals is that they can take into account past mean values and past observations in reflecting the mean parameter of the conditional negative binomial distribution, and can also characterize temporal dynamics in the volatility of patient arrivals.

Our in-sample and out-of-sample empirical results for patient arrivals at a large Spanish university hospital confirm that the INGARCH model yields better results that the Poisson model, particularly for the lower and upper quantiles of the forecasted distribution of arrivals. The fact that an INGARCH models yields a better fit for the extreme quantiles is particularly useful for management decision-making regarding resource allocation, both when a surge in arrivals may negatively affect healthcare, or when a drop in arrivals may render resources spare. Likewise, the variability of patient arrivals is well informed by INGARCH model estimations.

## Data Availability

Data is available on request.

## Footnotes

1 Although the original negative binomial parametrization in those studies does not reflect the conditional mean, we keep the dynamics of the conditional mean in order to link these extensions to the Poisson case (in line with [33].

2 We also considered a log-linear specification as in Agosto and Giudici [2], given that it allows for negative dependence. However, the log-specification yielded poorer results than the specification presented here – a result that can be explained by the positive correlations in our data (see Table 5. This evidence is available from the authors upon request.
